# Modality Matters: How Combining Handwriting and Typewriting Practice Improves Chinese Skills Performance for Chinese L2 Learning in China

**DOI:** 10.3390/jintelligence14070113

**Published:** 2026-06-23

**Authors:** Yuhan Guo, Yu Zhou, Jiaxiang Zhang, Yitong Luo

**Affiliations:** 1 Faculty of Education, Shaanxi Normal University, Xi’an 710062, China; 54101@snsy.edu.cn (Y.G.); luoyitonglyt@163.com (Y.L.); 2 School of Humanities, Shaanxi Xueqian Normal University, Xi’an 710100, China; 3 School of Chinese Studies·School of Central Asia, Xi’an International Studies University, Xi’an 710128, China; zhangjiaxiang@xisu.edu.cn

**Keywords:** handwriting practice, blended practice, Chinese skills performance, Chinese learning motivation, Chinese learning attitude, Chinese L2 learning

## Abstract

Chinese language practice, especially handwriting and typewriting practice, has always been a key method for mastering Chinese in Chinese L2 learning. However, current research on blended practice modalities that combine handwriting and typewriting remains insufficient. This study used a pilot study with 30 international students to compare the associative patterns of handwriting practice and blended practice (a sequential multimodal practice wherein handwriting instruction was followed by typewriting) on students’ Chinese skills performance, Chinese learning motivation, and attitude. Results indicated that students using the blended practice were associated with significantly better Chinese skills performance, as well as higher levels of motivation and more positive attitudes compared to those using only handwriting. Exploratory path analysis identified a notable direct association between practice modality and Chinese skills performance; however, the pathways through motivation and attitude were not statistically detectable. These findings suggest that Chinese language teachers may consider designing lessons that incorporate this sequential blended practice, which may support improvements in L2 Chinese performance, motivation, and attitudes. Furthermore, second language learners should actively apply this practice modality to improve their Chinese L2 learning performance.

## 1. Introduction

In the process of learning Chinese as a second language (L2), learners often need to build a complete system of Chinese language knowledge through practice (Lyu et al., 2021; P. P. Sun, 2020). Chinese language practice mainly includes two modalities: handwriting practice and typewriting practice (Liao, 2023; Q. Zhang & Min, 2019). Handwriting practice refers to learners directly writing Chinese characters with pen and paper, constructing and memorizing characters through fine motor skills, thereby deepening their memory and understanding of Chinese knowledge (Hsiung et al., 2017). Typewriting practice refers to using digital devices such as computers, tablets, and smartphones, as well as related software programs, to generate Chinese characters through keyboard input, thus engaging in Chinese character learning activities (Coss, 2025). This modality is characterized by its convenience, efficiency, and attractiveness (P. N. Zhang, 2021). Based on this, learners’ Chinese L2 learning, including listening, reading, and writing, is likely to be affected by the practice modalities, thus impacting their Chinese skills and learning experience (Bourgerie et al., 2022; X. Wang & Wang, 2024; Q. Zhang & Min, 2019). Although both handwriting and typewriting practice are common learning methods, there is currently a lack of research on the impact of blended practice modality combining handwriting and typewriting on learners’ Chinese L2 learning process and skills.

Numerous studies have shown that Chinese practice contributes to Chinese learning effectiveness because it transforms superficial knowledge acquired through lectures and reading into deeper internalization and effective transfer of knowledge through active repetition and application, thereby forming stable neural representations in the brain and ultimately significantly improving comprehensive Chinese application ability (Guan et al., 2011; Rejeki et al., 2025; Liao, 2023). Grounded in Embodied Cognition Theory and Self-Determination Theory (SDT), each modality has the potential to contribute. Specifically, handwriting practice is expected to enhance Chinese language performance through sensorimotor engagement and orthographic deepening, whereas typewriting practice is more likely to foster learning motivation and positive attitudes by reducing physical demands and satisfying learners’ needs for autonomy and competence. Despite the theoretical rationale for integrating modalities, most empirical studies have primarily focused on single-modality practice. Some studies emphasize handwriting practice (Cao & Perfetti, 2016; Coss, 2025; Q. Zhang & Min, 2019), highlighting its role in enhancing fine motor skills and deepening the structural understanding of Chinese knowledge, which is conducive to positive learning attitudes (Chang et al., 2024; Guan et al., 2011). Others focus on typewriting practice (Chee, 2024; Mohsen, 2022; P. N. Zhang, 2021), noting its capacity for immediate feedback and personalized learning paths that significantly improve learning efficiency and motivation (Liu, 2021; P. N. Zhang, 2021). Furthermore, existing research has identified differences between the two modalities in character recognition, memorization, and writing abilities (Coss, 2025; Lyu et al., 2021; Q. Zhang & Min, 2019).

Although Chinese practice is indispensable in Chinese L2 learning, current research on the differences between traditional handwriting practice and blended practice remains relatively limited. Crucially, the term “blended practice” itself lacks a consistent operational definition in the existing literature. While many studies conceptualize it as the interleaving of modalities within a single session (e.g., combining digital and traditional tools in one lesson), this study proposes a broader, curriculum-level conceptualization of blending (Graham, 2021). Drawing on curriculum integration theories, we define “blended practice” as the systematic integration of distinct instructional phases within a cohesive pedagogical unit. In this view, a practice qualifies as “blended” if it combines different modalities (i.e., handwriting and typewriting) over the course of an instructional program, regardless of whether these modalities are simultaneous or sequential at the micro level of individual lessons. This definition accommodates instructional designs where foundational skills are established in one modality before transitioning to another, reflecting a sequential form of curricular blending. In the present study, the blended practice condition was implemented sequentially, beginning with handwriting and followed by digital typewriting within the same instructional unit. Therefore, this study aims to fill this research gap by systematically examining the specific differences between handwriting practice and this newly defined blended practice in Chinese L2 learning, and exploring the associative patterns among the variables, thereby providing empirical insights and practical guidance for Chinese language teaching.

### 1.1. The Effects of Different Chinese Language Practices on Chinese L2 Learning

Chinese language practice has become a common method for second language learners in their Chinese L2 learning, referring to students using pen and paper or electronic devices to practice Chinese characters and their applications (Q. Zhang & Min, 2019). In Chinese language practice modalities, handwriting practice explores the writing process of Chinese characters with pen and paper, promoting a deeper understanding and expression of character structure (Lyu et al., 2021). Typewriting practice utilizes human–computer interaction to recognize Chinese characters and vocabulary, and their immediate feedback offers knowledge prompts and helps learners quickly identify and correct errors, thereby enhancing the effectiveness of Chinese knowledge construction (Liao, 2023). Therefore, Chinese language practice can facilitate the transformation of second language learners’ superficial knowledge into a deeper Chinese knowledge system, bringing positive impacts to Chinese L2 learning (Lyu et al., 2021; Q. Zhang & Min, 2019).

Previous research has shown that handwriting practice and typewriting practice influence second language learners’ Chinese skills performance. On the one hand, research has found that handwriting practice is more effective than non-motor practice in enhancing Chinese character learning, significantly improving recognition, memory, and writing skills (Hsiung et al., 2017; Rejeki et al., 2025). For instance, Hsiung et al. (2017) study found that handwriting activities enhance visual vocabulary recognition and promote character learning, while Chang et al. (2024) research highlights the close link between character writing and fine motor skills, which require precise control of stroke order and direction, leading to a deeper understanding of character structure and better writing ability. The advantages of handwriting practice mainly benefit from the synergistic effect of multimodal encoding. By integrating visual (observing the shape of Chinese characters), motor (writing actions), and proprioceptive (feeling the stroke path) information, it allows learners to perceive and process Chinese characters from multiple dimensions, constructing rich, three-dimensional, and stable representations of Chinese characters (Chang et al., 2024; Rejeki et al., 2025). Additionally, grounded in embodied cognition theory, handwriting practice transforms abstract Chinese character symbols into tangible physical experiences. This deep integration of body and cognition enhances learners’ understanding of the connotation and structure of Chinese characters, leading to more efficient and lasting learning (Lakoff et al., 1999; Chang et al., 2024). However, handwriting practice has also presented challenges. It involves both understanding the structure of Chinese characters and relying on motor memory, which can lead to excessive cognitive load. Cognitive load theory suggests that working memory has a limited capacity, allowing students to process only a certain amount of information at once (Sweller et al., 2011). Handwriting demands deep cognitive engagement, including precise control of stroke order, structure, and spatial arrangement (Chang et al., 2024). While multimodal sensory engagement can enhance character recognition and memorization, it may overly consume cognitive resources needed for applying learned information, ultimately reducing learning effectiveness (Rejeki et al., 2025; Sweller et al., 2019).

On the other hand, research has found that typewriting practice allows learners to concentrate on content expression instead of the written form of Chinese characters, enhancing writing complexity and accuracy (Coss, 2025; Fang et al., 2026; X. Wang & Wang, 2024). This effect is attributed to the fact that typewriting practice may rely more on phonological or component-based cues (such as Pinyin input) rather than the overall shape of the characters, thus reducing the cognitive load on students (Liu, 2021; P. N. Zhang, 2021). Additionally, the immediate and precise feedback from typewriting practice offers helpful knowledge support, facilitating learners’ recall of Chinese and allowing learners to concentrate more on pronunciation or word selection rather than the visual complexity of Chinese characters. This shift reduces the difficulty of character recognition and enhances Chinese learning efficiency (Lyu et al., 2021; Mohsen, 2022). Furthermore, typewriting practice allows learners to easily modify and edit, facilitating timely error correction and improving text quality and fluency (X. Wang & Wang, 2024). However, some studies have suggested that while typewriting enhances the efficiency of Chinese input and output, it lacks fine stroke movements, potentially leading to insufficient motor memory (Liao, 2023; Q. Zhang & Min, 2019). Additionally, its capacity to distinguish the visual features of Chinese characters may be inferior to handwriting, hindering deep memorization and internalization of character structures and resulting in a lack of depth in understanding (Chang et al., 2024; Rejeki et al., 2025).

Additionally, comparative studies indicated that while typewriting practice enhanced short-term vocabulary accumulation and grammar practice due to its efficiency, handwriting practice offered greater benefits for long-term vocabulary retention and deep understanding. Handwriting was particularly more effective in mastering the form, sound, and meaning of Chinese characters, especially in memorization and writing (Cao & Perfetti, 2016; Guan et al., 2011). Although typewriting practice excels in fluency (word count and speed), some studies suggested that handwriting might encourage learners to produce texts with more complex syntax and precise word choice (Farnè et al., 2025; Longcamp et al., 2008; Lyu et al., 2021). Thus, these two learning modalities differ in language production and Chinese learning outcomes, each with its own strengths.

### 1.2. The Effects of Chinese Learning Motivation and Attitude on Different Chinese Language Practices on Chinese L2 Learning

Chinese learning motivation is the sum of internal and external factors that drive learners to participate in and persist in learning Chinese, influencing their choice of learning strategies and ultimate learning outcomes (Csizér & Dörnyei, 2005; Dörnyei, 1994). Additionally, Chinese learning attitude is grounded in Expectancy-Value Theory (EVT; Eccles & Wigfield, 2020), which explains attitudes as shaped by expectancy for success and subjective task values. Consistent with this framework, Chinese learning attitude reflects learners’ level of interest, willingness to persist, and evaluation of Chinese learning activities (Douglass, 1977; Reid & Amanat Ali, 2020). Research has found that Chinese language practice has a unique and complex impact on Chinese learning motivation and attitude, with self-determination theory serving as the core theoretical framework for understanding this impact (Z. Chen et al., 2020; Deci & Ryan, 2004; Mangen & Balsvik, 2016). Numerous studies have shown that satisfying the three basic psychological needs of autonomy (feeling that the behavior is voluntary), competence (feeling capable of completing tasks), and belonging (feeling connected to others) is a key factor in stimulating learners’ intrinsic motivation and positive emotional experiences (Deci & Ryan, 2004; Ryan & Deci, 2000). Handwriting practice, being a highly embodied practice, may influence competence and autonomy through fine motor skills and deep cognitive processing (Mangen & Balsvik, 2016; Ryan & Deci, 2017). Typewriting practice, being an efficient and standardized practice, can influence learners’ sense of competence and belonging by lowering the operational threshold and providing immediate feedback, thereby affecting learners’ motivation and attitude towards learning Chinese (Z. Chen et al., 2020; Ryan & Deci, 2000).

Research has shown that handwriting practice has a dual effect on learning motivation and attitude. On the one hand, handwriting practice can have positive effects. Handwriting practice demands fine motor control. Learners’ deep engagement in completing complex tasks and improved motor accuracy fosters a strong sense of accomplishment, which boosts intrinsic motivation and encourages a positive learning attitude (Chang et al., 2024; Rejeki et al., 2025). Moreover, handwriting practice enhances learners’ focus and perseverance, further increasing their motivation and positive attitude towards the challenges of mastering complex Chinese characters (Guan et al., 2021; H. Sun et al., 2024). On the other hand, handwriting practice can have negative effects (Liao, 2023; X. Wang & Wang, 2024). For beginners, or those with poor fine motor skills, the cognitive load and skill demands of handwriting Chinese characters can cause initial frustration, undermining their sense of competence and triggering negative emotions like anxiety and fear. This may reduce their motivation and lead to avoidance of character writing (Q. Zhang & Min, 2019). Furthermore, handwriting practice is generally more time-consuming than typewriting, which can frustrate some learners and decrease their interest in learning, negatively affecting their motivation (Lu et al., 2019; P. N. Zhang, 2021).

Furthermore, research has shown that typewriting practice is remarkably effective, significantly reducing learners’ frustration, boosting motivation, lowering the difficulty of Chinese writing, enhancing students’ writing enthusiasm, and improving learning attitudes (Bourgerie et al., 2022; Liao, 2023; Q. Zhang & Min, 2019). However, the convenience of typewriting can create a “recognition–memory separation” phenomenon, where learners recognize Chinese characters but cannot write them, thereby hindering their deep processing of the characters’ form, sound, and meaning (Rejeki et al., 2025). Over time, this lack of solid knowledge may impair actual abilities and lead to instrumental motivation, such as learning solely to complete assignments, which can hinder the long-term development of intrinsic motivation (Liao, 2023). Moreover, as learning progresses, learners often neglect handwriting practice due to over-reliance on typewriting technology when encountering complex and multi-stroke Chinese characters. This behavioral pattern leads to obstacles in accurate character recognition and correct writing, weakening their interest in learning Chinese characters and ultimately resulting in a negative learning attitude (Chang et al., 2024; Rejeki et al., 2025).

In summary, when learning Chinese as a second language, handwriting practice and typewriting practice each have unique and complex effects on learners’ skill development, motivation, and attitudes, with their own advantages and limitations. Therefore, combining handwriting practice with typewriting practice to leverage their strengths is expected to create a complementary approach that may lead to better learning performance. However, research on how to effectively and scientifically combine these two modalities to enhance learners’ Chinese skills performance, motivation, and positive attitudes is still lacking. This study aims to explore the differences and associative patterns between handwriting practice and blended practice (a combination of handwriting and typewriting) regarding Chinese skills performance, motivation, and attitude. It seeks to provide practical guidance for Chinese teaching, optimize teaching methods, and enhance second language learners’ interest and enthusiasm for the language.

### 1.3. The Present Study

To fill the research gap regarding the impact of blended practice on Chinese L2 learning, we employed a pilot study at a foreign language university in China. This study aimed to explore the differential effects of single handwriting practice versus blended practice (a combination of handwriting and typewriting) on second language learners’ Chinese skills performance, Chinese learning motivation, and Chinese learning attitude, as well as to examine the associative patterns among these variables. Based on previous research, we formulated the following research questions and hypotheses:

RQ1. What are the differential effects of the two practice modalities on second language learners’ Chinese language skills?

**Hypothesis 1.** 
*Compared to handwriting practice, blended practice leads to better mastery of Chinese skills performance among second language learners.*

RQ2. What are the differential effects of the two practice modalities on second language learners’ Chinese learning motivation and attitudes?

**Hypothesis 2.** 
*Compared to handwriting practice, blended practice can inspire more positive Chinese learning motivation and attitudes among second language learners.*

RQ3. What are the associative patterns among practice modalities, Chinese learning motivation, learning attitude, and Chinese skills performance?

**Hypothesis 3.** 
*Chinese learning motivation and attitudes will show significant correlational pathways linking practice modalities to Chinese skills performance.*

## 2. Method

### 2.1. Participants and Research Design

This study recruited foreign students learning Chinese at a foreign language university in Northwest China. A total of 30 learners (11 males, 19 females; see Table 1) from two intact classes participated voluntarily. Participants were multinational, representing diverse cultural backgrounds, and a total of 12 nationalities were included in the sample, and their native languages were exclusively non-Chinese. Their mean age was 22.43 years (SD = 3.19). Regarding Chinese proficiency, eight participants held HSK Level 1 certification, while 22 held HSK Level 2 certification. Crucially, all participants possessed basic computer operation skills and were capable of typing in Chinese using Pinyin on smartphones, tablets, or computers. This baseline typing ability ensured that a lack of fundamental digital literacy did not confound the observed effects of the intervention.

We employed a pilot study with intact classes, assigning students to groups by class. The experimental class A of 15 students used a sequential multimodal practice (handwriting followed by typewriting) to complete the Chinese course content, while the control class B of 15 students used only handwriting practice. Both classes were taught by the same experienced Chinese teacher, and the teaching content was identical. To ensure instructional equivalence, the quantity and quality of practice were strictly matched across both groups, including the number of in-class exercises, homework assignments, and correction procedures; the only difference was the modality of output. It is worth noting that during the experiment, students in both classes did not participate in any other extracurricular Chinese L2 learning activities.

**Figure 1:**
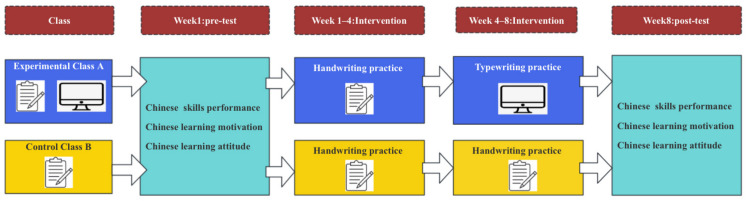
Experiment procedure.

This study design with a control group and pre- and post-test methods to examine the impact of different practice modalities on Chinese skills performance, Chinese learning motivation, and the attitude of second language learners when learning the same Chinese knowledge. The study was implemented in two stages, totaling 43 lessons (45 min each), over 8 weeks. Before the intervention, both classes underwent a pre-test to assess their initial Chinese skills performance, Chinese learning motivation, and attitude. During the intervention, the control group received 43 lessons of handwriting practice. The experimental group received a sequentially structured blended modality rather than an interleaved one. Specifically, the experimental curriculum was divided into two phases: Phase 1 included 22 lessons focused on handwriting, followed by Phase 2 with 21 lessons focused on typewriting. It is important to clarify that in this study, “blended practice” is defined as curriculum-level modality integration rather than intra-lesson integration. This sequential design was chosen because Chinese character learning typically begins with establishing orthographic-motor foundations (handwriting), after which digital input (typewriting) is introduced to support fluency and efficiency (P. N. Zhang, 2021). Therefore, although the modalities were implemented sequentially at the micro-level of individual lessons, they were integrated within the overall instructional unit, constituting a phased implementation of blended practice as defined in this study. After the intervention, a post-test was administered to assess learners’ Chinese skills performance, learning motivation, and attitudes. The researcher who administered the tests was not the course instructor; furthermore, anonymity was guaranteed, and participants were informed that responses would not affect academic grades. The procedure of the experiment is illustrated in Figure 1.

### 2.2. Measurements

#### 2.2.1. The Chinese Skills Performance Test

The Chinese skills performance test was developed by instructors in accordance with international curriculum standards and aligned with competency-based frameworks such as the HSK. “Chinese skills performance” was operationalized as a composite total score reflecting overall Chinese proficiency. To establish content validity, a panel of two senior Chinese L2 instructors (each with over 5 years of teaching experience) reviewed the items for relevance, curriculum representativeness, and linguistic appropriateness; items were revised based on expert feedback. The test was administered in paper format and consisted of parallel pre-test and post-test versions, each lasting 40 min. It comprised 25 items evaluating listening (8 items), reading (13 items), and writing (4 items). Writing responses were scored independently by two trained raters (senior Chinese L2 instructors with >5 years of experience) using a holistic rubric based on HSK writing criteria. Interrater reliability was high (ICC = 0.91); discrepancies were resolved through discussion, and the final writing score was computed as the average of the two ratings. All items were equally weighted to calculate the composite score, ensuring proportional representation of each skill domain.

To support the unidimensional structure of this composite score, an exploratory factor analysis (EFA) was conducted. For the pre-test, the KMO measure was 0.75, Bartlett’s test was significant (*p* < 0.001), the first eigenvalue was 1.91, and it explained 47.79% of the variance; factor loadings for the 25 items ranged from 0.56 to 0.74. For the post-test, the KMO measure was 0.76, Bartlett’s test was significant (*p* < 0.01), the first eigenvalue was 1.95, and it explained 46.62% of the variance; factor loadings ranged from 0.53 to 0.84. The test showed strong reliability, with Cronbach’s α = 0.82 (pre-test) and α = 0.84 (post-test). Given the modest sample size, the EFA results should be interpreted as preliminary. However, the unidimensional structure is consistent with the theoretically expected single proficiency construct underlying the HSK-aligned assessment.

#### 2.2.2. The Chinese Learning Motivation Scale

The Chinese character motivation scale was adopted from H. Chen’s (2007) adaptation of Lau’s (2004) Chinese Reading Motivation Questionnaire (CRMQ). Chen modified the original scale to assess motivation for Chinese character learning. Grounded in Organismic Integration Theory (OIT), the scale investigates participants’ motivation involved in character learning and contains four domains: intrinsic motivation, extrinsic motivation, social motivation, and self-efficacy, with four items per domain (16 items total). The items are scored on a 5-point Likert scale ranging from 1 (completely disagree) to 5 (completely agree). Higher scores indicate greater Chinese learning motivation. To validate the adapted scale in the current sample, an Exploratory Factor Analysis (EFA) was conducted using principal axis factoring with varimax rotation. The KMO measure was 0.76, and Bartlett’s test was significant (*p* < 0.001), indicating adequate sampling. EFA revealed a 4-factor structure consistent with the original theoretical framework, explaining 76.49% of the variance. All items loaded above 0.50 on their corresponding factors, with no cross-loadings exceeding 0.40. The scale demonstrated good reliability, with Cronbach’s α ranging from 0.70 to 0.85 across the four subscales (total α = 0.84). While the EFA supported the hypothesized structure, the small sample size limits definitive conclusions about factorial stability. The obtained factor pattern aligns closely with previous research using similar populations (H. Chen, 2007), supporting the instrument’s construct validity in this context.

#### 2.2.3. The Chinese Learning Attitude Scale

The Chinese learning attitude scale was adapted from Buschenhofen (1998) and Knezek and Christensen (1995), with modifications by H. Chen (2007). This scale evaluates participants’ attitudes toward character acquisition and comprises two domains: language attitude and computer attitude. These domains were conceptualized as interrelated components contributing to a unified construct of “Chinese learning attitude” in technology-mediated environments. Each domain has four items (12 items total), and the items are scored on a five-point Likert scale ranging from 1 (completely disagree) to 5 (completely agree). Higher scores indicate a greater Chinese learning attitude. To validate the adapted scale in the current sample, an Exploratory Factor Analysis (EFA) was conducted using principal axis factoring with varimax rotation. The KMO value was 0.77, and Bartlett’s test was significant (*p* < 0.001). The analysis yielded a two-factor solution accounting for 75.11% of the variance. All items loaded above 0.50 on their intended factors, with no cross-loadings exceeding 0.40. The reliability analysis indicated acceptable internal consistency. Specifically, Cronbach’s α was 0.76 for the language attitude subscale, 0.94 for the computer attitude subscale, and 0.85 for the overall combined scale. As with other measures, the EFA results should be considered exploratory due to the limited sample size. Nevertheless, the factor solution corresponds well with prior validations of the scale (H. Chen, 2007).

### 2.3. Data Analysis

The researchers conducted pre- and post-tests on Chinese skills performance, learning motivation, and attitudes during the first and last weeks of the course. A 2 × 2 mixed ANOVA was conducted with Time (pre-test, post-test) as the within-subjects factor and Practice Modalities (handwriting practice, blended practice) as the between-subjects factor. Furthermore, we utilized Model 4 of the PROCESS v4.2 macro in SPSS 27.0 to examine the associative pathways linking practice modalities to Chinese skills performance, with Chinese learning motivation and attitude included as parallel correlates.

Given the modest sample size (N = 15 per group, Total N = 30), we acknowledge the limitation in statistical power for detecting small effects. A priori power analysis using G*Power 3.1 (Faul et al., 2007) for a 2 × 2 mixed ANOVA (α = 0.05) indicated that the achieved power was approximately 0.52 for a medium effect size (f = 0.25). Although this power level is below the conventional 0.80 benchmark, it is sufficient to detect moderate-to-large effects, which are considered meaningful in exploratory educational interventions (Cohen, 2013).

Before conducting the 2 × 2 mixed ANOVA, the assumptions of normality (Shapiro–Wilk test) and homogeneity of variances (Levene’s test) were checked and met for all dependent variables (*p* > 0.05). Although the motivation and attitude scales used a five-point Likert, composite scores were computed as means. Treating these composite scores as approximately continuous is a common practice in applied linguistics research when distributional assumptions are checked (Field, 2024). To further address the small sample size, non-parametric Wilcoxon signed-rank tests (time) and Mann–Whitney U tests (practice modalities) were conducted as robustness checks; these analyses revealed that the Time main effect and Time × Practice Modalities interaction remained statistically significant (*p* < 0.05), consistent with the ANOVA findings.

## 3. Results

All analyses were conducted based on sample data comprising 30 students. This study performed a series of 2 (Time: Pre-test vs. Post-test) × 2 (Practice Modalities: handwriting practice vs. blended practice) Analyses of variance (ANOVA) to assess differences between the groups regarding Chinese skills performance, learning motivation, and learning attitudes. Descriptive statistics for the dependent variables are presented in Table 2.

### 3.1. RQ1: The Differential Effects of the Two Practice Modalities on Learners’ Chinese Skills Performance

**Figure 2:**
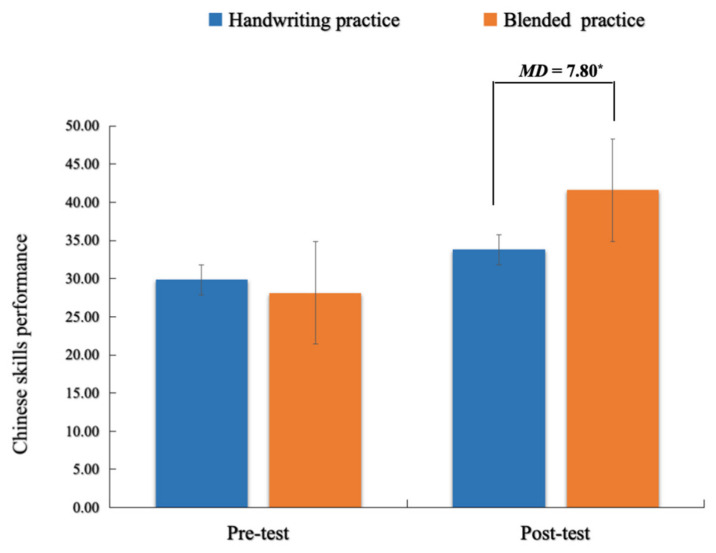
The interaction effect between times and practice modalities on Chinese skills performance. * *p* < 0.05.

A 2 × 2 mixed ANOVA was performed to investigate the effects of time and class on practice modalities (see Figure 2). The results revealed a significant main effect of time (F = 13.83, *p* < 0.001, η_p_^2^ = 0.198). In contrast, the main effect of class was not significant (F = 1.69, *p* > 0.05, η_p_^2^ = 0.029). Additionally, there was a significant interaction between time and class (F = 4.11, *p* < 0.05, η_p_^2^ = 0.068). Further analysis of simple effects indicated that there were no significant differences in Chinese skills performance among the two practice modalities during the pretest, suggesting that all students’ Chinese language skills were comparable before the intervention. However, following the intervention, the Chinese skills performance of students in the blended practice class (MD = 41.60) was significantly higher than that of the handwriting practice class (MD = 33.80). This finding provides partial support for H1. For more details, please refer to Figure 2.

### 3.2. RQ2: The Differential Effects of the Two Practice Modalities on Learners’ Chinese Learning Motivation and Attitude

**Figure 3:**
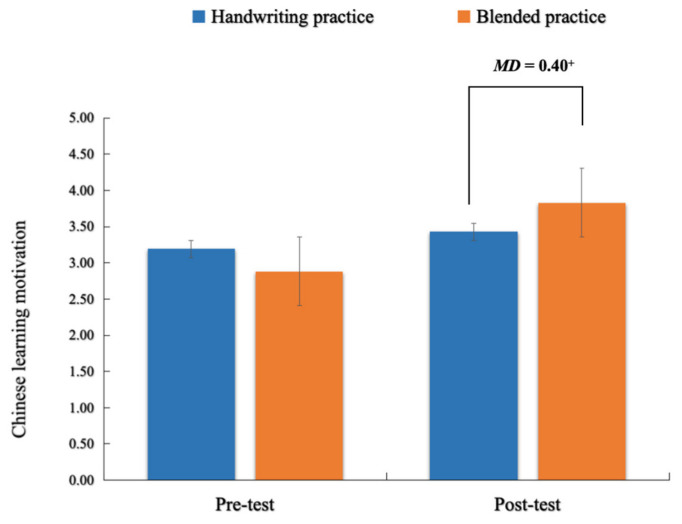
The interaction effect between times and practice modalities on Chinese learning motivation. 0.05 < ^+^ *p* < 0.1.

**Figure 4:**
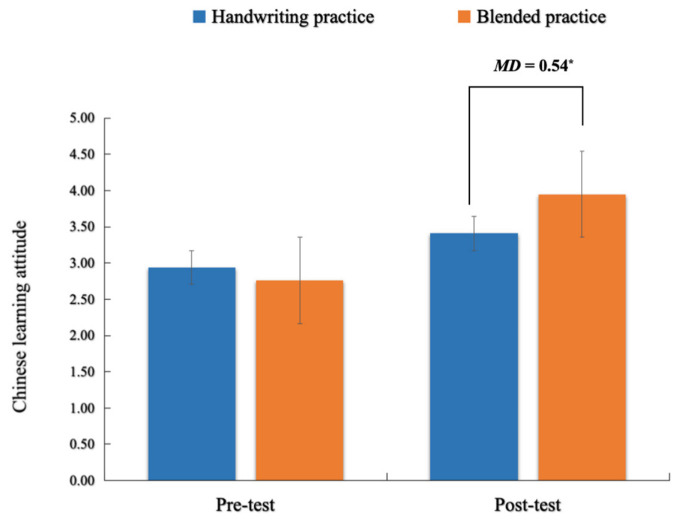
The interaction effect between times and practice modalities on Chinese learning attitude. * *p* < 0.05.

Figure 3 and Figure 4 display the results of the 2 × 2 two-factor mixed ANOVA for Chinese learning motivation and attitude. Firstly, regarding the effect of Chinese learning motivation, the analysis indicated a significant main effect of time (F = 16.72, *p* < 0.001, η_p_^2^ = 0.230), while the main effect of class was not significant (F = 0.09, *p* > 0.05, η_p_^2^ = 0.002). Additionally, there was a significant interaction between time and class (F = 5.80, *p* < 0.05, η_p_^2^ = 0.094). Further investigation of simple effects revealed no significant differences in Chinese learning motivation among the two practice modalities during the pretest, indicating that students’ motivation levels were comparable before the intervention. However, after the intervention, the Chinese learning motivation of the blended practice class (MD = 3.83) was marginally significantly higher than that of the handwriting practice class (MD = 3.43).

Secondly, the findings regarding Chinese learning attitude showed similar results to those for learning motivation. The main effect of time was significant (F = 21.51, *p* < 0.001, η_p_^2^ = 0.278), but the main effect of class was not significant (F = 0.99, *p* > 0.05, η_p_^2^ = 0.017). Furthermore, there was a significant interaction between time and class (F = 4.06, *p* < 0.05, η_p_^2^ = 0.068). Analysis of simple effects indicated no significant differences in Chinese learning attitudes among the two practice modalities during the pretest, suggesting that students’ attitudes were comparable before the intervention. However, the Chinese learning attitude of the blended practice class (MD = 3.95) was significantly higher than that of the handwriting practice class (MD = 3.41). This finding supports H2.

### 3.3. RQ3: Path Analyses of the Relationships Among the Practice Modalities, Chinese Learning Motivation, Attitude, and Chinese Skills Performance

To further explore the associations among variables, we examined whether Chinese learning motivation and attitude showed significant correlational pathways linking practice modalities to Chinese skills performance. The correlation relationships between the various variables are presented in Table 3. Notably, a significant correlation was found between Chinese skills performance and Chinese learning attitude (*p* < 0.05), as well as between Chinese learning motivation and Chinese learning attitude (*p* < 0.01). However, the correlation between Chinese skills performance and Chinese learning motivation was not significant.

We utilized Model 4 from the PROCESS macro for SPSS (Hayes, 2017) to conduct an exploratory path analysis examining the associative patterns among the variables. Practice modality was coded as a dummy variable (0 = handwriting practice, 1 = blended practice), with practice modality as the independent variable, Chinese learning motivation and attitude as parallel correlates, and Chinese skills performance as the dependent variable. Pretest scores for Chinese skills performance were entered as covariates to control for baseline differences. Variables were centered to mitigate potential multicollinearity. Given the small sample size (N = 30), bootstrapping with 5000 samples was employed; however, we acknowledge that this does not compensate for the limited statistical power to detect indirect effects.

**Figure 5:**
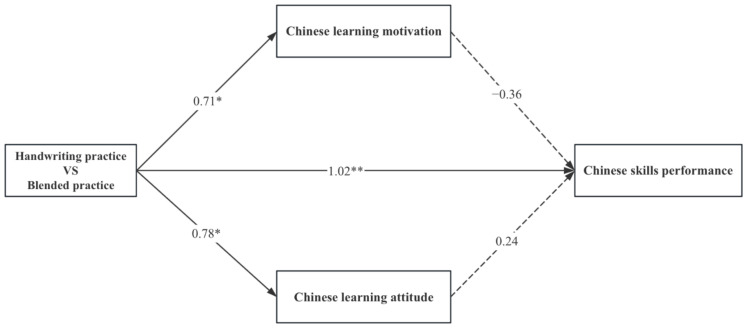
The path model. Path values are the path coefficients. ** *p* < 0.01, * *p* < 0.05.

Figure 5 illustrates the path model and standardized coefficients. The analysis indicated that blended practice was associated with higher observed levels of Chinese learning motivation (β = 0.71, *p* < 0.05), Chinese learning attitude (β = 0.78, *p* < 0.05), and Chinese skills performance (β = 1.02, *p* < 0.01) compared to handwriting practice. However, the paths from Chinese learning motivation (β = −0.36, *p* > 0.05) and attitude (β = 0.24, *p* > 0.05) to Chinese skills performance were not statistically significant.

Furthermore, the total effect of practice modality on performance remained significant when controlling for the correlates (B = 7.26, SE = 2.20, *p* < 0.01, 95% CI [2.74, 11.78]). The direct path of practice modality on Chinese skills performance was also significant (B = 7.74, SE = 2.60, *p* < 0.01, 95% CI [2.39, 13.08]). Conversely, the indirect pathways through motivation (B = −0.25, BootSE = 0.22, 95% CI [−0.84, 0.01]) and attitude (B = 0.19, BootSE = 0.15, 95% CI [−0.07, 0.52]) were not significant, suggesting that the data do not indicate meaningful mediating pathways via motivation or attitude in this sample. Therefore, H3 was not supported in the current exploratory model.

## 4. Discussion

### 4.1. Empirical and Theoretical Contributions

Chinese language practice is a commonly used strategy for second language learners and plays a crucial role in language acquisition. To investigate the differences between pure handwriting practice and blended practice (a combination of handwriting and typewriting), this study conducted a pilot study. It examined variations in Chinese skills performance, learning motivation, and attitudes among Chinese L2 learners. In addition, an exploratory path analysis was conducted to examine the associative patterns among practice modalities, Chinese learning motivation, learning attitudes, and Chinese skills performance. The results indicated that blended practice was more effective than pure handwriting practice in enhancing learners’ Chinese skills, stimulating motivation, and fostering positive attitudes. And the exploratory path analysis revealed that the relationships between practice modalities and performance were not mediated by motivation and attitude.

This study employed a two-way ANOVA to examine the differences in Chinese skills performance, Chinese learning motivation, and attitudes before and after the intervention between handwriting practice and blended practice. The results indicate that learners using the blended practice modality significantly outperformed those using handwriting practice alone, given consistent learning content (Mohsen, 2022; P. P. Sun, 2020; P. N. Zhang, 2021). This aligns with previous research on embodied cognition theory, suggesting that both handwriting and typewriting positively influence second-language learning in Chinese, and highlights the blended practice modality’s advantages in skills such as character recognition, reading, and writing (Lyu et al., 2021). From a cognitive perspective, the benefits of blended practice arise from the synergistic effects of both methods (Mohsen, 2022; X. Wang & Wang, 2024; A. Zhao et al., 2026). Specifically, handwriting practice, through multimodal cognitive processes, fully mobilizes multiple senses such as vision, touch, and kinesthetics, helping learners build stronger and more lasting Chinese language memories (Rejeki et al., 2025; Liao, 2023; Longcamp et al., 2008). Efficient typewriting practice facilitates extensive language output, improving fluency and accuracy (Hsiung et al., 2017; Liao, 2023). By combining these approaches, learners can express themselves effectively while developing a deeper understanding of Chinese character shapes, thereby mitigating issues related to a lack of comprehension in character structure that may arise from relying solely on typewriting (Chee, 2024; Wiley & Rapp, 2021).

Furthermore, findings indicated that learners in both the handwriting practice and blended practice groups showed improvements in motivation and attitude from pre- to post-test; however, the blended practice group exhibited significantly greater gains, aligning with previous research on the positive effects of multimodal practice (Chang et al., 2024; Xu & Hong, 2025; Q. Zhang & Min, 2019). This result supports the view that incorporating typewriting into L2 Chinese instruction may offer motivational and attitudinal benefits. Specifically, the superior outcomes observed in the blended group are consistent with the possibility of a synergistic effect, whereby the tactile engagement of handwriting and the efficiency and immediacy of digital input jointly support learner engagement. Although the present study did not directly measure cognitive load or metacognitive processing, prior literature suggests that such mechanisms may help explain these findings. For example, handwriting engages multiple sensory channels and fosters a sense of accomplishment when producing complex characters, potentially strengthening intrinsic motivation (Rejeki et al., 2025; H. Sun et al., 2024). Typewriting, by contrast, provides rapid feedback and personalized support, which may enhance learners’ self-efficacy and engagement (Bourgerie et al., 2022; Liao, 2023; Liu, 2021; P. N. Zhang, 2021). Moreover, blended practice may reduce the fatigue and frustration sometimes associated with slower or less legible handwritten production, allowing learners to express ideas more fluently—a pattern reported in studies linking multimodal input to reduced cognitive strain (Hsiung et al., 2017; Mohsen, 2022; Wiley & Rapp, 2021). While these interpretations remain speculative in the context of the current data, they offer theoretically grounded directions for future research examining the cognitive and metacognitive processes underlying blended L2 Chinese learning.

This study employed an exploratory path analysis to examine the associative patterns among Chinese practice modalities (handwriting practice vs. blended practice), Chinese learning motivation and attitude, and Chinese skills performance. The results revealed a significant association between practice modalities and Chinese skills performance; however, the specific indirect pathways through motivation and attitudes were not statistically significant. However, the specific indirect pathways through motivation and attitudes were not statistically significant in the current sample. This suggests that, within the scope of the current measurements, the relationship between practice modality and performance is more parsimoniously characterized by a direct association rather than an indirect pathway through these motivational factors. One possible explanation is that the effects of practice modalities may involve more direct cognitive or self-regulatory mechanisms than those captured by motivation and attitudes in the current study (H. Sun et al., 2024; Y. Wang & Yang, 2023). For instance, prior research suggests that blended practice may optimize cognitive resource allocation and reduce cognitive load (Sweller, 2010; H. Sun et al., 2024; Y. Wang & Yang, 2023). However, as cognitive load was not measured in the present study, its potential mediating role remains speculative. Additionally, blended practice may encourage learners to develop monitoring strategies related to metacognition (Razkane et al., 2026; M. Wang & Zhang, 2026). Future research should directly assess these constructs to determine whether cognitive load or metacognition mediates or moderates the effect of practice modality on performance.

### 4.2. Limitations and Future Directions

This study has revealed some findings on the impact of blended practice on Chinese language acquisition; however, it has several limitations that future research should address:

First, the internal validity of this study is limited by the intact class design. With only two classes (one per condition), the instructional modality (blended vs. handwriting) is completely confounded with class-level variables. Observed differences may stem from pre-existing disparities between the cohorts, peer dynamics, classroom environment, or other uncontrolled contextual factors, rather than solely from the practice modality itself. We acknowledge that this limitation cannot be fully resolved through statistical adjustments or interpretive reframing. Future research must employ randomized controlled trials (RCTs) with random assignment of individuals across multiple classes to establish definitive causal links.

Second, the sample size was relatively small (N = 30). While exploratory factor analyses (EFAs) were conducted to examine the factor structures of the motivation, attitude, and proficiency measures, these results should be interpreted as preliminary due to the limited sample size. Consequently, confirmatory factor analysis (CFA) was not performed, as CFA requires substantially larger samples to yield stable parameter estimates and reliable model fit indices. More importantly, the modest sample size resulted in insufficient statistical power to support the inferential strength of the path analysis. Although bootstrapping was employed to enhance the robustness of the estimates, it does not compensate for the fundamental lack of power required to detect significant indirect effects in mediation models. Therefore, the path model presented in this study is strictly exploratory and descriptive; no definitive claims regarding causal mechanisms or the statistical significance of mediation effects can be made. The absence of significant mediating pathways could be interpreted as a reflection of the underpowered design rather than conclusive evidence of no effect. Future replication with adequately powered samples is necessary to validate these exploratory findings and to test the hypothesized mediation effects formally.

Third, although prior literature suggests that cognitive load and metacognitive processing may underlie the observed effects, these constructs were not directly measured in the present study; thus, related interpretations in the Discussion are speculative and intended to generate hypotheses for future research. The operationalization of “blended practice” warrants further clarification. In this study, blended practice was implemented as a sequential transition from handwriting to typewriting rather than a truly integrated or interleaved modality within individual lessons. Consequently, it remains unclear whether the observed performance gains were driven by the synergistic effects of modality integration or by confounding factors such as the novelty of transitioning to typewriting or the specific sequencing of the curriculum. Future research should explore interleaved designs (e.g., alternating modalities within a single class) to disentangle these effects.

Finally, this study adopted a cross-sectional design, which prevents definitive conclusions regarding the causal sequence among practice modality, motivation, attitude, and performance. Accordingly, the path model presented is heuristic and exploratory, and future research employing longitudinal designs is necessary to establish true mediation effects.

### 4.3. Conclusions and Implications

This study innovatively integrates typewriting practice with handwriting practice to construct a blended practice modality and systematically examines its association effects in Chinese L2 learning. Empirical analysis results show that, compared to single handwriting practice, the blended practice modality was associated with significantly better Chinese skills performance, as well as higher levels of learning motivation and positive attitudes. However, further exploratory path analysis revealed that motivation and attitudes did not exhibit significant correlational pathways linking practice modalities to performance. These findings suggest that the observed advantages of blended practice may operate through mechanisms other than general motivation or attitude.

Despite replicating some expected patterns, this study makes several contextual and integrative contributions to the literature on Chinese L2 learning. First, by embedding both handwriting and typewriting within a coherent curriculum-level blended design, the study moves beyond the common “single-lesson” conceptualization of blended practice. This clarifies how blended modalities can be sequenced across instructional units to support the acquisition of Chinese characters. Second, the study provides a joint test of embodied cognition and Self-Determination Theory (SDT) within the same dataset, offering a crucial clarification: while embodied motor engagement may benefit performance directly, motivational pathways do not necessarily mediate this effect. This pattern challenges assumptions often made in L2 instructional research. Third, from a practical standpoint, the findings suggest that a well-structured blended practice modality can yield skill gains. This offers a feasible and scalable alternative for Chinese language programs operating under time or resource constraints. These findings carry important implications for instructional design. Rather than assuming universal motivational benefits, educators should recognize that the advantages of blended practice may stem primarily from direct cognitive and pedagogical efficiency. This insight helps optimize Chinese L2 intervention strategies under real-world constraints.

## Figures and Tables

**Table 1:** Basic information about Participants.

	Category	Number	Frequency (%)
Gender	Male	11	36.67
	Female	19	63.33
Nationality	Russian	5	16.67
	Kazakhstani	5	16.67
	Japan	5	16.67
	Uzbekistan	4	13.33
	South Korea	3	10.00
	France	2	6.67
	Bosnia	2	6.67
	Vietnam	1	3.33
	Germany	1	3.33
	North Macedonia	1	3.33
	Morocco	1	3.33
HSk	1	8	26.67
	2	22	73.33
Class	Experimental Class A	15	50.00
	Control Class B	15	50.00

**Table 2:** Means of all dependent variables, with standard deviations in parentheses.

Variable	Time × Practice Modalities			
	Pre-Test and Handwriting Practice	Pre-Test and Blended Practice	Post-Test and Handwriting Practice	Post-Test and Blended Practice
Chinese skills performance	29.83 ± 10.58	28.13 ± 11.47	33.80 ± 7.98	41.60 ± 4.72
Chinese learning motivation	3.19 ± 0.53	2.88 ± 0.57	3.43 ± 0.57	3.83 ± 0.60
Chinese learning attitude	2.94 ± 0.81	2.76 ± 0.61	3.41 ± 0.75	3.95 ± 0.58

**Table 3:** Correlation analysis results of the variables.

	M	SD	1	2	3
1 Chinese skills performance	33.34	10.28	1		
2 Chinese learning motivation	3.33	0.65	0.24	1	
3 Chinese learning attitude	3.26	0.82	0.30 *	0.56 **	1

Notes. * *p* < 0.05, ** *p* < 0.01.

## Data Availability

The data used to support the findings of this study are available from the corresponding author upon request.
